# Awareness of ADHD in primary care: stakeholder perspectives

**DOI:** 10.1186/s12875-020-01112-1

**Published:** 2020-02-28

**Authors:** B. French, E. Perez Vallejos, K. Sayal, D. Daley

**Affiliations:** 1 Division of Psychiatry & Applied Psychology, University of Nottingham, Nottingham, England; 2 UK & Centre for ADHD and Neurodevelopmental Disorders Across the Lifespan (CANDAL), Institute of Mental Health, University of Nottingham, Nottingham, England

**Keywords:** ADHD, Interviews, Primary care, Pathway to care

## Abstract

**Background:**

Attention Deficit Hyperactivity Disorder (ADHD) is underdiagnosed in many European countries and the process of accessing care and diagnosis is complex and variable. In many countries, general practitioners (GPs) refer on to secondary care where individuals receive an assessment and, if appropriate, a diagnosis and access to care. It is therefore essential that GPs have a clear understanding of the disorder and its care pathways. While previous studies have highlighted potential barriers in GPs’ ADHD awareness, this qualitative study aims to further explore individual stakeholders’ experiences.

**Methods:**

Semi-structured interviews explored the views of multiple stakeholders- GPs (*n* = 5), healthcare specialists (*n* = 5), patients (adults with ADHD *n* = 5) and parents (*n* = 5) with experience of the presentation and management of ADHD in primary care. These interviews were analysed using thematic analyses and following principles of grounded theory.

**Results:**

Stakeholders described ADHD assessment, diagnosis and treatment as an intricate process. Many factors affected this process such as complex pathways, lack of services, limited GP recognition and knowledge, and communicative difficulties between and within multiple stakeholders.

**Conclusion:**

This analysis underlines the significant impact that receiving (or not) a diagnosis can have, and further explores muddled ADHD care pathways, highlighting key issues around GP identification and the shortage of adult services. Implications for practice and future research are discussed, suggesting a strong need for more commissioned pathways and GP specific educational programs.

## Background

Attention deficit hyperactivity disorder (ADHD) is a neurodevelopmental disorder affecting 3–5% [1] of children, with symptoms often continuing into adulthood. While popular beliefs suggest that ADHD is an over diagnosed condition this belief is a point of contention amongst researchers [2, 3], and strong evidence exists indicating that ADHD is being underdiagnosed in many countries worldwide [4–6]. In the UK, for example, reports suggest that only 0.73% of children and 0.06% of adults receive ADHD medication [7]. Even when patients have received a diagnosis, medication use varies widely across European countries [8], with medication use in the UK being relatively low. Issues experienced by people with ADHD in childhood can lead to considerable cognitive and behavioural impairment [9, 10], affecting social behaviour, school work and family life [11, 12]. In adulthood, these issues are associated with higher rates of criminal behaviour, loss of work, addiction, suicidality and failed relationships [13]. While evidence-based treatments have been shown to help manage ADHD symptoms [14], untreated ADHD can have strong economic and social burdens [15]. There is therefore a strong need for early detection and diagnosis.

Typically, the first port of call in gaining an ADHD diagnosis in the UK is through primary care and General Practitioners (GPs). While referrer eligibility can vary across different service providers, in the majority of cases, GPs act as gatekeepers and after identification will then refer on to secondary care services - Paediatric or Child and Adolescent Mental Health Services (CAMHS) for children, or Adult Mental Health Services - where individuals can gain an assessment, a diagnosis and access to treatment if required. GPs are also often responsible for handling prescriptions of medication once treatment has been initiated. Limited recognition by GPs of ADHD has been shown to be a key barrier in accessing diagnosis and treatment [16] with many GPs reporting low levels of confidence in the recognition and management of ADHD [17]. A recent systematic review [18] examined barriers related to GPs understanding and recognition of ADHD and highlighted four main issues: i) need for education (lack of training and knowledge), ii) lack of resources (time and financial), iii) presence of misconceptions, and iv) a need for a multidisciplinary approach. These issues present a challenge to GPs’ recognition of ADHD and consequently may impact on their willingness and ability to refer for an assessment and diagnosis.

Qualitative interviews with healthcare practitioners have helped to highlight specific issues experienced in ADHD referral such as viewing the diagnosis process as inherently complex [19] and requiring time and experience [20]. Gaining an understanding of stakeholders through individual interviews from multiple perspectives will help us to gain a better understanding of individuals’ experiences and difficulties within primary care. The present qualitative study aims to further explore the primary care experience of referral and management of ADHD. In order to gain this deeper insight, semi-structured interviews were conducted with GPs as well as other key stakeholders directly involved in the primary care pathway and ADHD diagnosis, specifically parents of children with ADHD, adults with ADHD, and secondary care workers who assess and treat people with ADHD.

## Method

### Study design

Semi-structured interviews were conducted over a three-month period in late 2018 with participants from across the UK. The interviews were conducted by the lead investigator (BF) who has received extensive training in qualitative methods and were analysed using thematic analysis [21].

### Participants

Nineteen participants were interviewed for the purpose of this study, representing the views of twenty individuals. One participant explored issues related to diagnosis both as a parent and as an adult patient as her son’s diagnosis triggered her own referral and diagnosis. The participants were selected from four different stakeholder groups: i) GPs, ii) secondary care professionals who specialised in ADHD diagnosis, iii) adults with ADHD, and iv) parents of children with ADHD. These groups of participants were carefully selected in order to give a representative sample of the stakeholders directly involved in ADHD diagnosis, integrating patients and professionals’ perspectives. Participants were interviewed in no specific order to limit biases from specific groups and were each given a monetary inconvenience allowance for their participation.

#### GPs

Three males and two females GPs were interviewed (mean age: 33y.4 m, range: 44y.7 m-29y.4 m). The GP participants were recruited from the local Clinical Research Network and through direct contact to practices.

#### Adults with ADHD

Nine participants gave consent but only five took part in the study. Two male and three female adults with ADHD were interviewed (mean age: 48y.8 m, range: 63y.3 m-29y.2 m) from across the UK. The adults were recruited from ADHD adult support groups known to the lead investigator.

#### Parents

Five female parents of children with ADHD were interviewed (mean age: 41y.2 m, range: 62y.10 m-29y.5 m) from across the UK. The parents were recruited from parenting support groups known to the lead investigator.

#### Secondary care professionals

Three male and two female secondary care specialists from the UK were interviewed (mean age: 46y.5 m, range: 63y.5 m-36y.6 m). Two participants worked with adult patients and dealt with adult diagnosis. Three participants worked in child diagnosis settings (two in CAMHS and one in a community paediatric team). These participants were selected purposely to represent secondary care workers both with adults and children.

### Data collection

Individual semi-structured interviews were conducted to explore GPs attitudes and understanding of ADHD. After the participants were made familiar with the interview process, written consent was obtained. Participants were offered a choice of telephone or face to face interviews. All except one took place over the telephone. The use of these different data collection methods had no impact on the data analyses as both interview methods reflected similar themes, which has been shown by previous research on the comparability of the two methods [22]. Three interview schedules (one for GPs, one for patients and one for secondary care professionals) were developed based on a recent literature review [18] and included specific topics as well as more open ended questions (Supplementary Material 1). The three interview schedules covered the same topics but from different standpoints according to participant group. GPs were asked a greater number of specific questions as this group was our main focus of interest. Following a grounded theory approach [23] the interview schedule was applied flexibly and reviewed on a regular basis with data analysed on a continual basis throughout the process. Questions were changed or added as different topics emerged. Certain questions were also omitted depending on the participant’s experience. Detailed notes were taken and recorded after each interview and following each analysis and included in an analysis diary. All interviews were audio recorded and transcribed verbatim and all transcripts were anonymised.

### Data analysis

The analytic strategy for this study was based on thematic analysis [21] using an inductive approach, enhanced by the principles of grounded theory [24]. Themes and subthemes were identified using an adapted approach of Braun and Clarke’s [20] six stage process. While previous literature reviews [18, 25] and a pilot study highlighted topics that needed to be explored, the interview schedule was developed in a way that allowed new topics to emerge in an inductive manner, aiming to freely explore the participants’ experiences. The analytic process began by transcribing each interview verbatim, shortly after being conducted. Following this process, the lead investigator first familiarized herself with the interviews by listening to the audio tapes and reading through the transcripts a number of times. Following verbatim transcription, the lead investigator took notes in a diary of her preliminary thoughts on the content of the interviews. From this close familiarisation with the transcripts, preliminary codes were identified in a coding manual. After familiarisation with these codes, they were then collated and combined to be classified into broader themes using constant comparative analysis both within and between transcripts. Finally, as the analysis evolved, theses broader themes were reviewed and refined and generated the final themes proposed. In order to get a meaningful analysis, it was ensured that data within each theme was coherent in relation to each theme and subtheme as well as within the context of the overall dataset. Ongoing analysis allowed for a clear definition of the final themes.

Themes were finally reviewed by a second researcher (EPV) to ensure that they mapped to the original transcripts. The second reviewer also confirmed that theoretical saturation was reached and that no new themes emerged in the last few interviews, as suggested by thematic analyses guidelines and studies with similar methodologies [26, 27].

The coding manual and theme extraction were checked by the second researcher along with the individual coding of transcripts. Inter-rater reliability was tested on a small proportion (10%) of the transcripts’ themes and sub-themes. The results were validated collectively as a team, and any discrepancies were discussed and reconciled.

## Results

The identified codes highlighted five main themes (Table 1). While some themes overlapped strongly with previous findings from literature reviews [18], new topics emerged from our synthesis.

The new topics highlighted by this analysis were: GPs’ not identifying ADHD and the lack of services and pathways to care. These concepts were present within all interviews and reflected by all stakeholders. These concepts were seen to impact on other themes.

**Table 1:** Main themes and subthemes identified

Themes	Subthemes
Lack of identification in primary care	No identification in primary care Patient-led approach and strategies
Lack of clear diagnosis pathway and services	Complexity of services Long waiting lists and triage Age specific issues
GPs’ knowledge of ADHD and misconceptions	Insufficient knowledge and complex role Misconceptions
Impact of diagnosis and the risks linked to no diagnosis	Impact of a diagnosis on the patients GPs’ negative view of ADHD label
Difficult communication between multiple stakeholders	Communication between services Communication with patients

### Lack of identification in primary care

#### No identification in primary care

The main theme highlighted by this analysis, related to the concept of identification of ADHD. Specifically, GPs indicated that they were not the ones identifying ADHD symptomatology when faced with patients’ issues. All interviewees, when asked who was responsible for picking up ADHD agreed that it principally came from the patients. Only one GP described identifying ADHD in his patients, and then only around 10% of the time. All other GPs acknowledged that they had never identified ADHD in a patient. It was more common for patients to raise the issue of ADHD with their GPs, with identification of symptoms being triggered by personal reflection or a third party, something referred to as “chance diagnosis”. Third party identification was often triggered either by schools or through the diagnosis of a first degree family member (child or sibling).

“My experience has always been a parent has brought their child in saying, “I want a referral to paediatrics. I think my child has ADHD.” It’s either the school suggesting it to the parents or the parents suggesting it” (P11- GP)

“ADHD was not picked up, not for many years […] my wife picked it up, my wife who works in a school […] but the doctors never picked it up” (P2- adult)

“I was looking into it for my sons and then realised that actually I’ve probably got it myself” (P4- adult)

#### Patient-led approach and strategies

Patient-led approaches have implications both for the initiation of referral, and the subsequent process of referral. As the process is usually not led by the GPs, patients stated that as well as having to ask for a referral and initiating an ADHD enquiry, they also had to push to get a diagnosis. Strength of character and stubbornness were key factors in getting through the process and patients believed that without a constant effort on their part, they wouldn’t have received a diagnosis.

“So yeah, there was basically nothing on their part, it was just me pushing for it and me being proactive about it” (P5- adult)

“They need to say, what can we do to help you? That was never done. I’m lucky because I’m strong and feisty and I knew that there was something wrong” (P13- parent)

In order to address the lack of recognition by the GPs, patients developed strategies to bypass GP gatekeeping. For instance, some patients sought private diagnosis in order to access care. In particular, private diagnosis was sought when patients felt that they had reached a dead end, or strongly needed access to care, or perceived that GPs lacked awareness and were not acknowledging their issues.

“We didn’t want to wait, so we paid to see a private guy and he did the diagnosis very straight forward and very quickly for us” (P12- parent)

One patient explained that after years of issues, she had to “trick her GP” into giving her a referral for a diagnosis.

“No one had picked it up[…] So it was only when I read something online that sounded like me, and then did some further research and then tricked my GP into giving me a referral” (P5- adult)

While patients revealed being affected by issues surrounding lack of identification, this experience was something that GPs also acknowledged. GPs expressed that the identification of ADHD was a very complex process, and difficult to pick up in a consultation.

“So, we rely a lot on what parents tell us and parental concern as well. If we see the child, we’ll only have a brief interaction with the child, so our impression of the child is mainly based on history and parental concern [...] it comes from the parents mainly” (P14- GP)

“I don’t think I’ve ever had anybody come and say, there’s this, this and this and I’ve said, I think that’s ADHD” (P15- GP)

### Lack of clear diagnosis pathway and services

The next theme identified in the interviews related to the issues around diagnosis pathways and services. Despite clear guidelines, diagnosis pathways vary considerably between different areas. This is due to distinct commissioning priorities between healthcare localities (NHS Trust), which result in resources being allocated differently and a consequent impact on services. All interviewees agreed that their experience of diagnosis and management depended strongly on the services provided and the pathways in place. A ‘good’ service was perceived as one in which pathways were clear, communication facilitated and management processes were relatively straightforward. However, in most cases, services and pathways were reported to be very unclear, muddling the referral, diagnosis and management process.

#### Complexity of services

The complexity of the services was discussed at different levels, firstly through lack of service availability, secondly though GP’s lack of knowledge about what services were available and thirdly through variations in services depending on geographical areas. The lack of service availability greatly hindered the diagnosis and management process

“It can be difficult to get somebody assessed for ADHD […] So in my experience, I have had to send somebody out of area in the past in order that they can get a diagnosis or get some help and support for it” (P6- GP)

“So parents sometimes come to us and say they have waited a long time to see us and I’ve never really been sure why they’ve waited” (P18- secondary care professional)

“There isn’t a pathway because it’s not a commissioned service” (P19- secondary care professional)

“It was tough, there was nowhere for me to go to get a diagnosis or to see anyone who could give me a diagnosis” (P4- adult)

It was also discussed that even when there were services, the services were not known about or were often changing, making the process of referral confusing.

“So I think some GP’s may not even know that we (specialist service) exist actually” (P1- secondary care professional)

Finally, the referral process was often so complex that GPs had to refer to different services according to many variable factors, including geographical location, making it very difficult to keep track of which pathways they were supposed to follow.

“So I’ve tried referring them to the paediatricians locally here and I’ve had it bounced. I’ve referred them to the psychiatrist and I’ve had them bounced. I’ve asked is there a community and mental health team that can see this patient, and they say it doesn’t cover their remit. So I find it to be a very difficult referral process” (P7- GP)

“Because we’re a tertiary service and we don’t have the resource to be able to case hold and so case holding needs to take place in secondary care, not adult mental health services […] we don’t accept referrals from the GPs […] Because there’s conflict between the GPs and secondary care about who takes on the prescribing, so the area prescribing have not managed to reach agreement to develop a shared care protocol” (P19- secondary care professional)

#### Long waiting lists and triage

This lack of services and clear pathways had strong consequences on the referral process, principally creating long waiting times. With services overloaded due to limited resources, patients and professionals felt very frustrated by the excessive delays they often experienced.

“After waiting a year and a half to actually get an appointment at the ADHD clinic” (P10- adult)

“It does take a long time (to get a diagnosis). There is a very slow process and we’re trying to look at ways of making it better” (P9- secondary care professional)

In response to the significant delays and overloaded services, many specialist services have set up a system of triaging, putting in place different strategies such as stricter referral criteria or extra layers of screening or information gathering in order to manage waiting lists. These approaches aim to optimise scarce resources but risk potentially losing patients due to the long waits or to stricter criteria which may not get to the root of the problem.

“Services are either not funded or they’ll see people who fit very specific criteria and I know there is no management service” (P6- GP)

“Actually that is partly a deliberate off-putting tactic to try and reduce referrals, which is a terrible thing to say, but I’m sure that’s part of the motivation that it’s another obstacle to this flood of referrals that we get.” (P1- secondary care professional)

#### Age specific issues

The lack of services had different implications depending on whether it related to children or adult referral pathways. With children, issues related to overloaded secondary care services were often mentioned. This included difficulties with medication management and the difficulties getting hold of specialist services in a timely manner. This had a direct impact on GPs as patients felt that GPs should be able to take over when other services are overbooked.

“(with regards to medication) CAMHS they’re overloaded and understaffed […] GP surgery is far more accessible than trying to see a mental health professional out of your specified appointment time. So the GP can prescribe, but all he does is sign off on scripts, he can’t see them with regards to meds” (P12- parent)

“I think they should be able to contact CAHMS to talk about medication, ‘cause you can’t always get hold of CAHMS because the mental health system is so stretched, so the only other point of call you’ve got is your GP” (P16- Parent)

The main issue with regards to adults’ diagnosis, was the nonexistence of services. Most GPs mentioned that their area had no adult services and they therefore did not know where to refer adults to. This lack of commissioning in turn impacted the few existing services with extra referrals and therefore more delays.

“My experience of referral is only with children because there isn’t an adult service here in L.” (P11- GP)

“like I say then when you get to adults and there isn’t that kind of support around, effectively you’re giving them a diagnosis and you’re not able to do anything for them” (P15- GP)

In relation to the lack of adult services, issues with children transitioning from children to adult services were also raised. In these instances, not knowing who takes care of these individuals was a worry for all professionals. Having no transition services in place implied that GPs might have to carry on managing these individuals with no training or competence.

“When a child turns 18 and they’re no longer… they’re discharged from paediatrics but there’s no adult follow up. There is no pathway at all at the moment, everything just seems to stop” (P11- GP)

“I think probably one of the issues we’re going to be having is that as kids come out of paediatric care and they’re still on these medications, who is taking responsibility and I think at the moment it just defaults to the GP, basically” (P15- GP)

### GPs’ knowledge of ADHD and misconceptions

#### Insufficient knowledge and complex role

GPs’ limited knowledge of ADHD was often discussed throughout the interviews. It was felt overall that GPs were helpful and open to the idea of ADHD, however, the general consensus from all participants was that while they had some knowledge, they didn’t know enough. GPs generally felt they knew enough so that when ADHD was first mentioned, they were happy to refer on to specialist services, yet not enough to identify ADHD or give clear information on pathways and services. This concept of not knowing enough was expressed by healthcare professionals, GPs and patients alike. It was also acknowledged that there has been a general increase over the last decade in GPs’ understanding and awareness of ADHD. However, GPs were aware of their own limitations.

“So no, I feel like we’re very much in the dark when it comes to it and it’s a shame because we are usually the first port of call for parents when they’re concerned about this. I think there definitely is a lot room for improvement in this area” (P14- GP)

“General knowledge has really improved over the last 15 years […] Most know what they don’t know, if that makes sense. So if they don’t know what to do, they know to refer into specialists.” (P3- secondary care professional)

“He (the GP) had an understanding of it but was quite open and he would say “Okay, I will pass you on to the people that know about this more” (P4- adult)

The limitation of GPs’ knowledge mainly related to the process after referral, directly impacting both patients and specialist services. GPs did not know enough on pathways to diagnosis and management. Patients reported feeling frustrated as they had no information on the next steps after referral.

“No mention of any kind of support except for private support that was far too expensive” (P8- parent and adult)

“They put us on a waiting list with no other help or assistance and after a couple of years she went to be assessed” (P13- parent)

From a specialist standpoint, many secondary care workers reported that the lack of sufficient information received from the GPs meant that many referrals had to be sent back or it created longer delays.

“The problem was that some of the referral letters are so brief that there isn’t anywhere near enough information” (P1- secondary care professional)

This was discussed in terms of the lack of clear understanding of differences between ADHD and ASD. As these diagnoses can have different referral pathways, confusing them implies greater delays and/or the refusal of referrals.

“It’s like ASD and ADHD […] I get the impression that GPs don’t really know what either of these things are” (P1- secondary care professional)

The role of the GP in ADHD diagnosis and management is rather complex and this often created confusion for GPs and patients alike. GPs felt that they were not sure about their role, and that they would like to be able to give more support to their patients but didn’t have the relevant information.

“There’s a mismatch between an expectation of my role as a GP and what secondary care think we can and can’t do” (P6- GP)

#### Misconceptions

GPs’ knowledge was also discussed in relation to misconceptions. Stigmas around ADHD were still at times expressed, with the stigma of the “naughty child” often mentioned. One secondary care worker reported that one GP surgery in their area did not believe ADHD was a valid diagnosis. But this instance seemed to be the exception rather than the norm and a change in the last decade around more accurate understanding of ADHD and less stigma around ADHD was noticed. Rather than stigmas per se, broader misconceptions were expressed.

“So some peoples’ GPs tell them that only children get it, although that’s less often now” (P1- secondary care professional)

The main misconception related to the concepts of social economic status (SES) and parenting. These topics were often brought up by GPs as causal factors of ADHD. Parents expressed that they felt their parenting was questioned during the diagnosis process and GPs mentioned that they sometimes wondered if seeking a diagnosis was used as an excuse for bad parenting.

“Sometimes a feeling, almost of the parents are letting their child stay up really late, giving them fizzy drinks, sugary snacks, they’ve got all this sugar and fuelling the hyperactivity” (P6- GP)

“Is it ADHD or is this just bad parenting[…]because their parents either want a diagnosis for financial benefit or they feel like if I give my child a diagnosis it absolves me of the fact of parenting” (P7- GP)

“I was made to feel a little bit like it was my parenting discipline, which I was very upset about ‘cause I’ve been a qualified nursery nurse and a nanny for, like, over 20 years, so I found that quite insulting” (P16- parent)

GPs felt that SES was a strong risk factor and that they had biased views on patients from lower SES, expecting them to seek diagnosis more often. The biases stem from a strong belief that diagnosis is sought to gain access to welfare benefits. Colleagues’ opinions with regards to this specific misconception had an impact on GPs’ beliefs, some GPs acknowledging that colleagues’ mentalities strongly impacted their biases towards patients from lower SES seeking an ADHD diagnosis.

“However, there is also in my mind whether that is a bit of prejudice on my part and the medical professions part, that we’re almost looking for these problems in people of lower socioeconomic means which, if we saw perhaps a very affluent middle class parent with a child, we might not necessarily jump to that conclusion” (P6- GP)

“When I see individuals, unfortunately who are trying to con the system, and not only do I see this but I have my colleagues in my general practice come to me to say, another one trying to get her child a diagnosis. So I don’t think it’s just my personal bias but it’s also the practice bias” (P7- GP)

“It seems like a lot of parents who are saying ‘I think my kid has ADHD’ are generally of a lower socio-economic class and may be single mums and maybe have lots of children and maybe their life is a bit chaotic[…] maybe, asking for an explanation or an excuse in poorer families” (P15- GP)

GPs also had misconceptions around individuals’ behaviour in consultation. They stated that even though it doesn’t affect their final decision on referral, the patients’ behaviour strongly impacted their beliefs with regards to whether the patient might have ADHD.

“I’ve had people ring me up and say, this person says they’ve got ADHD but they sat beautifully still and concentrated well for the whole eight minute consultation?” (P3- secondary care professional)

“So, sometimes the parents will describe the child in a certain way and you think, oh my goodness, when this child comes he’s going to be bouncing off the walls […] Then they come in, they sit on the chair and they’re quiet, they’re polite, they’re okay and then you think to yourself, this doesn’t sound like the child that mum was describing earlier on. So sometimes it makes it a little bit difficult to marry that up” (P14- GP)

Finally, the last misconception related to ADHD in high-functioning adults and in girls. High functioning individuals and girls seem to go under the radar as they often do not meet the GPs’ beliefs about ADHD. They might be less hyperactive, less disruptive in class and therefore do not fit some conceptions attributed to ADHD.

“So anybody coming in as an adult is obviously not going to have really typical, really severe symptoms otherwise he would have been picked up or you know” (P15- GP)

“So, I think typically that stigma still exists for us, because GP’s, professionals, even teachers will say, actually they’re a quiet inattentive young girl rather than loud noisy boy. They can’t have ADHD because they’re not shouting at me or causing a problem in the class, or they can’t have ADHD because they’re not running around […] So I think there’s still that thought that if you’re not extreme, you don’t have difficulties warranting a psychiatric assessment” (P3- secondary care professional)

### Impact of diagnosis and the risks linked to no diagnosis

#### Impact of a diagnosis on the patients

The positive impact of receiving a diagnosis was discussed by patients and secondary care workers. The benefits of receiving a diagnosis with regards to gaining access to care and gaining a greater understanding of individuals’ issues was often a great help and relief for the individuals.

“(upon receiving a diagnosis) I was relieved and I think he (her son) was relieved […] I think he welcomed it. He was self-medicating a lot on drugs and not going down a very good route at all, and since he has been on the medication he’s not really touched drugs very much” (P8- parent and adult)

While gaining a diagnosis was linked to many positive outcomes, adult patients who had all received a diagnosis in adulthood, felt a lot of mixed emotions upon receiving an ADHD diagnosis. Receiving a diagnosis opened many doors and was an overall positive experience, yet frustration and anger were also expressed that this had not been picked up earlier. Adult patients felt a sense of loss and missed opportunities for the years they spent undiagnosed and expressed that they wished it had been identified sooner.

“I felt a bit annoyed really because I would have liked to have known way back, earlier than that. It came as a big shock… […] what worries me is that many people are put on the wrong drugs, wrong medication when it isn’t being picked up” (P2- adult)

“But I’m still cross… we’ve wasted years really” (P13- parent)

The delay experienced in receiving a diagnosis also had other negative implications for the adult patients. Some adults self-medicated with drugs or alcohol before seeking a referral and also while waiting for their diagnosis in the absence of other coping mechanisms. Some patients stated that they sought a diagnosis when they were experiencing severe issues and the additional wait led to distressing feelings, depression, time off work and at times could lead to risk-taking behaviours.

“So then people wait for 18 months to two years at the moment, which I think is not uncommon, but it’s very hard for them and for us really because we just know that they aren’t going to improve in that time and it may lead to lots of life problems […] at times it can be life threatening, if people do stupid things or feel suicidal and so on” (P1- secondary care professional)

“So typically we see teenage girls who come into the CAMHS service for self-harm or overdosing. They’re very frustrated with their life, they’re suffering educationally, something happens and their skills to be able to cope with things implodes or they just kind of struggle and do self-harm or something like that” (P3- secondary care professional)

“and I had to get to that stage where I felt I was in desperate need ‘cause I was just being passed around from pillar to post and if I hadn’t have been quite strong, sort of thing, I can see how some people in that position would do something silly and would harm themselves […] and I tell you what, I drank a hell of a lot of alcohol and self-medicated on other things” (P4- adult)

“The whole thing was quite upsetting if I’m being honest” (P16- parent)

#### GPs’ negative view of ADHD label

While patients and secondary care workers expressed many benefits in gaining a diagnosis, GPs on the contrary expressed negative bias to the diagnosis of ADHD, wondering why patients would want this diagnostic label.

“Some GPs are very reluctant to make a label or a diagnosis because of stigma attached to it […] I’m consciously aware that it’s a diagnosis that’s probably not very nice for people to have” (P6- GP)

They also expressed that they did not see the point of seeking a diagnosis in adulthood given that adults had somehow managed so far. The ADHD label was linked with strong negativity from the GPs and they struggled to see the positives associated with it in adulthood.

“I think I definitely wonder sometimes, as an adult, is this going to change anything for you? It’s the case with any investigation we do or any referral, you’re giving somebody a label. A diagnosis, is it actually helpful?” (P15- GP)

### Difficult communication between multiple stakeholders

The last theme identified from the interviews referred to issues with communications. The lack of clarity in the communication between services created more work, more delays in the processes. This encompassed both difficulties with communication between and within services (primary care and secondary care) but also communication between services and patients.

#### Communication between services

The complexity of the diagnostic process meant that communication was often very difficult between services with a general confusion about their designated roles. From the GPs’ perspective, the lack of services and change in referral pathways resulted in GPs not knowing where to refer to and referrals being sent back. They also were unsure of the different information they were supposed to send and which services to refer to.

“You give them all the information, you think, wow this seems like it’s really good information but then they’ll write back and they’ll say they don’t necessarily think it’s an appropriate referral and things like that […] so it would be nice if there was a little bit more of a way to communicate with community paediatrics” (P14- GP)

The nature of an ADHD diagnosis meant that a lot of information from different stakeholders need to be gathered in order to proceed. Waiting for information to be sent back from schools, patients etc. also created longer delays both for primary and secondary care services and the communication through these processes was also at times difficult.

“It can be a very quick process or it can be a very strenuous process depending on the school” (P3- secondary care professional)

“There was supposed to be a system set up where schools gave an awful lot of information to the GPs to pass on to the paediatricians and for some reason that doesn’t happen” (P9- secondary care professional)

From the specialist services, the lack of accurate information, or not enough information from the GPs in general, meant that they struggle to know how to proceed with diagnosis for specific referrals.

“We had a bit of a problem in that GPs were not giving some of the information that we needed, some of the letters were minimal […] At the referral stage, it’s a bit frustrating for both sides really. So if they send me a letter and I think, oh I don’t really know what I need to know, I’ve sent it back to the CPE, the CPE have said to them fill in this form, then they send me the form which is a bit of a hold up” (P1- secondary care professional)

“In terms of primary care, it varies considerably because every GP practice, as you can imagine, has a different admin system and so some are much more efficient than others” (P18- secondary care professional)

#### Communication with patients

Patients received very little information about ADHD following referral, both with regards to the diagnostic process and the management process. Many reported that once the referral had been sent through, they had no idea about how long it would take, what the process involved, and what was to come next. This implied a lack of communication both from primary and secondary care services.

“I asked for a call back and didn’t get that. So eventually I made an appointment with my GP who referred me back to the ADHD clinic and that got lost as well, so eventually I had to call the clinic again” (P10- adult)

“So there wasn’t clear communication between them and me either, so I filled in a questionnaire to get onto the waiting list and I didn’t hear anything. I assumed that they decided I didn’t have anything, they weren’t going to give me an appointment and then all of a sudden, 18 months later out of the blue, I got an appointment letter to go and visit them” (P5- adult)*.*

“We were left with this big bombshell, and not; “If you need help in the meantime you can contact various agencies in your area.” It was, “Nope, see you in four months, but I’ll give you a ring in a month to see how you’re getting on with the medication.” (P16- parent)

Patients felt that services were unwilling to take responsibility and lead the process with clear communication. One of the main issues expressed by the patients was a feeling of being constantly passed around. With one service telling them to go to another and vice versa. Patients reported feeling dismissed and wondering why there was such a reluctance to provide information on the process.

“So, unbelievably frustrating, there just aren’t the resources there and you just ended up getting passed from pillar to post and you got pushed onto someone else, and someone else, and someone else […] I felt it was a stacking system, you were being stalled ” (P4- adult)

“The school kept telling me to go to the GP, the GP said no, they can’t refer us, the school had to. I was like a ping pong ball, you know, going backwards and forwards” (P17- parent)

## Discussion

This thematic analysis yielded many inter-related themes from multiple perspectives on ADHD awareness in primary care in the UK, primarily focusing on issues with pathways, identification and communication. The findings have the potential advantage of including standpoints from multiple stakeholders involved in the diagnosis and management process of ADHD, highlighting many similarities in their experiences of ADHD care.

The qualitative nature of this investigation allowed for a strong focus on participants’ own experiences and for more targeted topics to be discussed from a stakeholder perspective. A recent quantitative study investigating GPs’ attitude and knowledge towards ADHD [28] found that very few GPs had a positive attitude towards ADHD. While this was discussed in our interviews, our study allowed this topic to be explored further, emphasising specific difficulties with communication and misconceptions that is harder to capture in a quantitative format.

Our findings also strongly overlapped with previous research. In a recent systematic review [13] considerable lack of accurate knowledge, issues with services and difficult communication between multiple stakeholders were also found to be barriers to access to care for ADHD. Semi structured interviews conducted with UK and Belgium clinicians [20] investigating decision making in the management of ADHD also reported issues around multidisciplinary communication and the lack of clear, operationalised guidelines and services. Finally, GPs and parent interviews on barriers to treatment of hyperactivity [29] also highlighted issues with pathways to care, misconceptions, GPs lack of experience and knowledge.

GPs often act as gatekeepers to accessing care and without their referral it is often impossible to access diagnosis or treatment. It was therefore very interesting to find that the main topic highlighted by this study was the lack of identification from GPs. This reflected previous findings on GPs’ non-recognition being a principal barrier in the pathway to care [30]. While no patient or GP stated that GPs had ever refused or interfered with the referral process, the ADHD referral process is often a patient-led approach strongly based on self-education and awareness. Implications for patients who have no awareness of ADHD are consequently compelling [31]. If a patient does not know about ADHD or is not aware of the wide spectrum of ADHD symptomology (inattentive type versus hyperactive type for instance), they might never seek a diagnosis or receive appropriate access to care. GPs stated that they also never had a referral refused. While this was interpreted as a very low diagnosis threshold, this is more likely to mean that the nature of a patient-led approach means that a wide range of patients may be missed and ADHD may be often under-diagnosed.

The second difficulty relating to ADHD awareness is specific to the UK healthcare system and covered the complexity and lack of clear pathways for children and adults’ services, these services varying widely across the country. Moreover, as the ADHD referral and diagnosis process involves multiple stakeholders (school, families, secondary care, etc.), this increases the complexity of the communication between them as it requires a number of different individuals to appropriately respond. This was also highlighted by the concept of shared care agreements where GPs are able to take over the prescription of ADHD medication. These agreements are not compulsory and vary widely between practices but without them, patients have to go to overbooked secondary care services, making the process more complex and lengthy.

Waiting times were also an important topic highlighted in these interviews often with a negative connotation. GPs reported not knowing how to support their patients during the long wait and patients reported symptoms and mood worsening overtime. Secondary care workers also reported feeling upset knowing patients had to wait a long time and not enjoying having to find ways to triage patients due to the ever growing waiting lists. All stakeholders felt frustrated and helpless at addressing this particular issue. As patients reported years of struggle before being aware of their diagnosis, and at times having only looked into ADHD once they had reached a crisis point, the extra time added to access care was felt to be very damaging to patients. While waiting times were discussed in the interviews, all participants were asked about their own experience with delays, both in seeing a secondary care worker and in receiving a diagnosis. A recent study [32] investigated diagnosis times in Europe and found that the UK had the longest waiting time (on average 18.3 months) from first visit to the GP to a formal ADHD diagnosis. They also reported that the UK time from first noticing symptoms to a formal ADHD diagnosis was on average 31.9 months. These findings reflect strongly the views expressed in this study, with great delays in accessing care but also, due to the patient-led approach, further delays between first noticing symptoms and accessing care, at times of up to almost 3 years.

### Strengths and limitations

Having four different groups of participants in this study provided a more holistic approach to the understanding of the referral process, allowing for multiple stakeholder perspectives to be taken into consideration. While the different groups had different experiences, the overarching themes were mostly expressed by all groups, indicating a strong relevance of the issues presented. This relevance was also reinforced by the fact that themes overlapped with previous published research in the literature.

The findings presented by this study are of international relevance for countries where GPs hold a gatekeeping role in ADHD identification and referral [6] having strong implications for practice and research.

It is important to note that while this report reflects key concerns from multiple stakeholders’ experiences, these are based on their own individual experiences and practices and might not necessarily map onto other stakeholders’ experiences.

The majority of GPs taking part in this study (4/5) were a self-selected sample of young GPs, newly qualified (within 5 years). While they expressed a strong interest in ADHD, they might have had limited experience in referral. The input of older GPs who might have had more experience in seeing ADHD patients is lacking. It is also the case that GPs who qualified over a decade ago might also be less likely to have received ADHD training. Secondary care professionals who had greater experience of ADHD noticed a changed in ADHD awareness in the last decade. One participant stated that he delivered training to GP trainees annually and therefore knew that all GPs in his region did receive some ADHD training. Therefore, it could be assumed that younger GPs might have been more likely to have received training on ADHD and therefore have a better awareness of the disorder.

A few interesting points arose from our stakeholder sample. For instance, no fathers were represented. Only mothers took part in this study which limits our analysis by not including a paternal view. Similar studies have found that mothers’ views tend to be reported much more often in the literature than fathers’ [33, 34]. This could be potentially explained by the cultural implication of mothers often being the one taking their child to the GP. However, two males were represented in our adult patient sample.

### Implication for practice

These interviews have demonstrated that GPs are ill equipped to identify and manage ADHD in primary care, in part due to barriers in access to care, lack of knowledge and resources, lack of clear pathways and services. These factors created discomfort around the process of diagnosing and supporting patients with ADHD. Our findings indicate a need for increased and more specific awareness training about ADHD, clearer pathways and more services to be commissioned in order to support the ongoing delays experienced in ADHD diagnosis and treatment, with a greater focus on adult services and transitioning patients. Better integration between primary and secondary care services may also address issues with communication, further support for GPs, and promote better services. Further training on ADHD identification and awareness could also reduce GPs’ uncertainties about ADHD. Finally, support during the diagnosis process is strongly needed, providing management strategies through the lengthy diagnosis process.

### Implication for research

This study provides a deeper insight into the primary care experiences of ADHD, both from a GP perspective but also from other groups involved in ADHD diagnosis and management. A clear lack of knowledge and understanding was presented in this study and future research should focus on addressing these issues. By increasing accurate knowledge and reducing misconceptions, validated psychoeducational interventions on ADHD - tailored specifically to GPs - could address these issues. This study also potentially opens further exploration into how these findings might generalise more widely to other psychiatric disorders.

### Open educational resource

An open educational resource has been developed taking into consideration the issues raised in this study along with previous findings on a literature review [18]. This resource was co-designed with GPs in order to tailor it to their needs. This resource aims to enhance awareness and understanding of ADHD and clarify the role of primary care in ADHD diagnosis and treatment.

This resource is available at www.adhdinfo.org.uk and is distributed under a creative commons license. It contains interactive tools, testimonies videos of patients, specialists and GPs.

## Conclusions

In conclusion, this study highlights a strong need for early diagnosis and for better identification from GPs. Many barriers prevent this from happening and while some are difficult to address, such as the complexity of the diagnosis pathway in the UK, others can be addressed by better awareness and education on ADHD.

## Supplementary information

**Additional File 1.** Interview Schedule.

## Data Availability

The datasets used and/or analysed during the current study are available from the corresponding author on reasonable request.

## Abbreviations

ADHD: Attention Deficit Hyperactivity Disorder
GP: General Practitioner
