# Supplementary material for: Awareness of ADHD in primary care: stakeholder perspectives
Source: BMC Fam Pract. 2020 Feb 28;21:45. doi: 10.1186/s12875-020-01112-1 (PMC7047346; doi:10.1186/s12875-020-01112-1)
Supplement: Supplementary file 1 — Additional File 1. Interview Schedule. [file 12875_2020_1112_MOESM1_ESM.docx]

Interview Schedule secondary care ADHD awareness

Topics to be discussed in interview

Can you tell me a little bit about your experience of referral and diagnosis of ADHD?

Relationship between primary care and secondary care

- What are your beliefs and understanding about ADHD
- Do you think your colleagues in primary care share your views
- Do you think primary and secondary care professionals have similar understandings? Do you think primary and secondary care professionals talk the same language?
- Is there clear communication between primary and secondary care?
- Does it take a lot of time for information to be passed around?
- Do you feel that there is a clear pathway between all disciplines to ADHD diagnosis and treatment?

Misconceptions

- As a professionals have your ever been aware of stigmas or misconceptions about ADHD to patients or parents?
- Did you witness any stigma/misconceptions by any other professionals during consultations or team meetings?
- How important do you think patient behavior is during the consultation is, in helping to reinforce concerns about possible ADHD?

Need for education

- Do you feel GPs know a lot/enough about ADHD?
- Do you think GPs have relevant information on the nature of ADHD, treatment and or diagnosis process?
- Who first mentioned the possibility of ADHD (GP, teacher, parent?)
- Do you think GPs have enough information to refer to secondary care (teacher’s report, parents interview, child behaviour in office)

Speed of process

- How long does it take to get a referral from primary care from first enquiry?
- How long does it take to get a diagnosis?
- What would you like GPs to know or to do differently before referring ADHD patients to secondary care?

Anything else you would like to add?

Interview Schedule patients ADHD Awareness

Topics to be discussed in interview

Can you tell me a little bit about your experience of referral and diagnosis of ADHD?

Relationship between professionals

- During the diagnosis process, do you think there was clear communication between primary and secondary care?
- Did it take a long time for information to be passed around?
- Do you think primary and secondary care professionals shared your beliefs and understanding about ADHD?
- Do you think primary care professionals had similar beliefs about ADHD?
- Do you feel that there is a clear pathway to get an ADHD diagnosis and treatment? What was it like for you?

Presence of misconceptions

- Did your GP ever mention any stigmas or misconceptions about ADHD? Can you tell me a little bit more about them if they did?
- Did you witness any stigma/misconceptions by any other professionals during the diagnosis process?
- Did any professional mentioned causes such as parenting or society?
- Do you feel your/your child’s behaviour during consultation impacted the diagnosis process?

Need for education

- Did you find your GP knew a lot/ enough about ADHD?
- Did your GP give you information on the nature of ADHD, treatment and or diagnosis process?
- Who first mentioned the possibility that your child/ you may have ADHD (GP, teacher, parent?)
- Which criteria/information did the GP ask from you before referral to secondary care (teacher’s report, parents’ interview, child behaviour in office).

Speed of process

- How long did it take to get a referral to secondary care from first enquiry?
- How long did it take to get a diagnosis?

Anything else you would like to add?

Interview Schedule GP’s ADHD Awareness

Topics to be discussed in interview

Can you tell me a little bit about your experience of referral and diagnosis of ADHD?

Relationship between primary care and secondary care

- What are your beliefs and understanding about ADHD
- Do you think your colleagues in primary care share your views
- Do you think primary and secondary care professionals have similar beliefs and understandings
- Is there clear communication between primary and secondary care?
- Does it take a lot of time for information to be passed around?
- Do you feel that there is a clear pathway to ADHD diagnosis and treatment between primary care and secondary care?

Presence of misconceptions

- As a professional, are you aware of any stigmas or misconceptions about ADHD?
- Did you witness any stigma/misconceptions by any other professionals during the consultation process?
- Are you aware of having any negative connotations associated with the label ADHD?
- What role do you think parenting plays in the development and maintenance of ADHD?
- How important do you think patient behavior is during the consultation is, in helping to reinforce concerns about possible ADHD?
- What role do you think society or SES plays in the development and maintenance of ADHD?

Need for education

- Do you feel as a GP, you know a lot/enough about ADHD?
- Do you have relevant information on the nature of ADHD, treatment and or diagnosis process
- Who first mentioned the possibility of ADHD (GP, teacher, parent?)
- Which criteria/information do you use before referral to secondary care (teacher’s report, parents interview, child behaviour in office)?

Speed of process

- How long does it take to get a referral to secondary care from first enquiry?
- How long does it take to get a diagnosis?
- Do you feel adequately equipped to deal with such diagnosis?
- Once a child get a diagnosis of ADHD, does that mean extra work for you?

Would you welcome more education and training about ADHD?

What format would they like for intervention: paper, website, short clips daily or one 45 minutes etc.

Will it make a difference in their taking part if it was CPD?

Would you like to take part in second stage of the study?

Anything else you would like to add?
